# Distribution of Thrombophilia-Related Genetic Polymorphisms in Women with Reproductive Disorders

**DOI:** 10.3390/biomedicines14010199

**Published:** 2026-01-16

**Authors:** Almagul Kurmanova, Madina Khalmirzaeva, Nagima Mamedalieva, Gulfiruz Urazbayeva, Damilya Salimbayeva, Damira Ibrayeva, Alfiya Dzheksembekova, Zhanar Kypshakbayeva, Altynay Nurmakova, Elif Salar

**Affiliations:** 1 Department of Obstetrics and Gynecology, Faculty of Medicine and Healthcare, Al-Farabi Kazakh National University, Almaty 050040, Kazakhstan; almagul.kurmanova@kaznu.edu.kz (A.K.); mamedalieva_kz@mail.ru (N.M.); elf_ernazarovna@mail.ru (A.D.); kypshakbaevazhanar@gmail.com (Z.K.); elifsalar44@gmail.com (E.S.); 2 Department of Strategic Development and Science, Scientific Center for Obstetrics, Gynecology and Perinatology, Almaty 050010, Kazakhstan; gulffa@mail.ru (G.U.); sdamilya@mail.ru (D.S.); nur_altinay01@mail.ru (A.N.); 3 Department of Surgical Diseases, Faculty of Medicine, Khoja Akhmet Yassawi International Kazakh-Turkish University, Turkestan 161200, Kazakhstan; damira.esey@mail.ru

**Keywords:** gene polymorphism, thrombophilia, early pregnancy loss, fetal loss syndrome, preeclampsia, postpartum hemorrhage, antiphospholipid syndrome

## Abstract

Thrombophilia is considered one of the key mechanisms underlying reproductive disorders. Clinical heterogeneity of reproductive disorders and a lack of stratification by phenotype often limit interpretation. Therefore, evaluating thrombophilia-associated genetic markers separately in fetal loss syndrome, postpartum hemorrhage (PPH), and hypertensive disorders of pregnancy is essential. **Background/Objectives**: To assess the frequency of thrombophilia-related genetic polymorphisms in women with various reproductive disorders and evaluate their association with clinical–anamnestic characteristics and obstetric antiphospholipid syndrome. **Methods**: A total of 132 women with reproductive disorders (fetal loss syndrome, postpartum hemorrhage, preeclampsia). **Results**: Statistically significant differences were found when comparing between the groups. Thus, heterozygous *F13* genetic polymorphisms were statistically more common in the group with a history of preeclampsia compared to the group with PPH (the G/A genotype was detected in 22.2% versus 10.7%, *p* = 0.045), and heterozygous *ITGA2* gene genetic polymorphisms were also more common (the C/T genotype was detected in 66.7% versus 42.9%, *p* = 0.023). In women with a history of PPH, homozygous *ITGA2* genetic polymorphisms were statistically more common (the T/T genotype was detected 2.6 times more often—21.4% versus 8.8% compared to the group with fetal loss syndrome, *p* = 0.022; and 3.8 times more often—21.4% versus 5.6% compared to the group with PE, *p* = 0.022). **Conclusions**: A study of thrombophilia gene polymorphisms in women with reproductive disorders showed that the G/A genotype of F13, the C/T genotype of ITGA2, and the A/G genotype of MTR:2756 were significantly more common in women with preeclampsia than in the group with postpartum hemorrhage; the T/T genotype of the ITGA2 gene was detected in postpartum hemorrhage. The MTHFR 1286A > C (A/C) polymorphism was associated with a reduced risk of postpartum hemorrhage. In contrast, the MTR 2756A > G (A/G) genotype was associated with an increased risk of preeclampsia.

## 1. Introduction

Thrombophilia is considered one of the key mechanisms underlying reproductive disorders. Hereditary or acquired defects of anticoagulant proteins, coagulation factors, fibrinolytic components, and platelet receptors lead to an imbalance in hemostasis and increased thrombotic tendency. Clinically, thrombophilia manifests through venous and arterial thrombosis, fetal loss syndrome, preeclampsia, placental insufficiency, postpartum hemorrhage, and other pregnancy complications [1,2,3,4].

Population-based and family studies indicate that 40–60% of individual susceptibility to venous thrombosis is genetically determined [1,5,6]. The most extensively studied genetic polymorphisms include prothrombin (*F2 G20210A*) and factor V Leiden (*F5 G1691A*), predominantly found in Caucasian populations [2,7]. Other important variants include polymorphisms in *F7*, *F13*, *FGB*, *PAI-1*, platelet receptors *ITGA2* and *ITGB3*, as well as folate-cycle enzymes (*MTHFR*, *MTR*, *MTRR*), which influence homocysteine metabolism and endothelial function [3,8,9,10].

In obstetrics, attention has shifted not only to classical thrombophilias but also to their interaction with the antiphospholipid syndrome (APS). Antiphospholipid antibodies promote endothelial dysfunction, complement activation, and microthrombosis, contributing to severe preeclampsia, HELLP syndrome, placental insufficiency, fetal growth restriction, and adverse pregnancy outcomes [4,11,12,13,14].

Despite numerous studies, the contribution of individual genetic polymorphisms to obstetric complications remains inconsistent, especially in Asian populations [10,15,16]. Clinical heterogeneity of reproductive disorders and lack of stratification by phenotype often limit interpretation. Therefore, evaluating thrombophilia-associated genetic markers separately in fetal loss syndrome, postpartum hemorrhage (PPH), and hypertensive disorders of pregnancy is essential.

In this regard, the aim of the study was to assess the frequency of occurrence of genetic polymorphisms associated with thrombophilia in women with various reproductive disorders and to evaluate their relationship with clinical and anamnestic characteristics and obstetric antiphospholipid syndrome.

## 2. Materials and Methods

### 2.1. Subjects

This was a retrospective study involving patients with reproductive disorders who underwent testing for gene polymorphisms. A total of 132 women with reproductive disorders were included. According to their obstetric-gynecological history, they were divided into three groups:

Group 1: fetal loss syndrome (FLS) (*n* = 68), women with recurrent miscarriages, missed miscarriage, or intrauterine fetal demise, specifying ≥2 consecutive pregnancy losses in accordance with ESHRE and ACOG guidelines [17,18,19].

Group 2: postpartum hemorrhage (PPH) (*n* = 28), defined per WHO and FIGO criteria [20,21].

Group 3: preeclampsia (PE) (*n* = 36), diagnosed according to ISSHP and ACOG recommendations [22,23].

The inclusion criterion: all women had ≥2 previous pregnancy losses, reflecting a high probability of thrombophilic or autoimmune mechanisms [24,25]. Evaluation for sexually transmitted infections (STIs) was performed according to ESHRE and WHO recommendations [18,26].

APS status was assessed based on prior testing (LA, anticardiolipin antibodies, anti-β_2_GP1). Positive results confirmed APS according to ACR/EULAR 2023 and Sydney 2006 criteria [27,28,29]. In women without prior APS testing, non-criteria antiphospholipid antibodies (anti-PS, anti-annexin V, anti-prothrombin, etc.) were evaluated using an expanded panel, consistent with contemporary evidence on non-criteria aPL in obstetric APS [30,31,32].

### 2.2. Sample Preparing

Peripheral venous blood was used as the biological material. Blood samples were applied to special FTA cards, dried at room temperature for 2–3 min, and then stored at room temperature.

### 2.3. Genetic Testing

Genetic studies were performed at Microread Technology Kazakhstan Co. Ltd. (Astana, Republic of Kazakhstan). Polymorphisms in genes associated with thrombophilia and folate metabolism disorders were analyzed using polymerase chain reaction (PCR) to assess their contribution to the pathogenesis of reproductive disorders and pregnancy complications. Genotyping was performed using the Thrombophilia Genetic Detection Kit (Beijing Microread Genetics Co., Ltd., Beijing, China) based on fluorescent PCR and capillary electrophoresis [33,34].

Eleven thrombophilia-associated genes were studied (Table 1): coagulation and fibrinolysis: *F2*, *F5*, *F7*, *F13*, *FGB*, *PAI-1*; platelet receptors: *ITGA2*, *ITGB3*; folate-cycle enzymes: *MTHFR*, *MTR*, *MTRR*.

### 2.4. Ethical Approval

The study was approved by the Local Ethics Committee of Al Farabi Kazakh National University, Kazakhstan (Code: IRBA902/IRB 00010790). All participants provided written informed consent for the use of biomaterials in this study.

### 2.5. Statistical Analysis

Statistical analysis was performed using IBM SPSS Statistics version 19.0. Quantitative data are presented as mean and standard deviation (mean ± SD), while categorical data are presented as absolute and relative values. Given the different sample sizes and heterogeneity of variances, the Welch *t*-test was used to compare quantitative indicators between groups (with the “equal variances not assumed” option). Frequencies of genotypes were shown as absolute values (*n*) and percentages (%). Categorical variables were analyzed using the Pearson χ^2^ test or Fisher’s exact test for low expected frequencies. In case of detection of statistically significant differences between groups, the odds ratio (OR) and its 95% confidence interval (CI) were calculated. The statistical significance was set at *p* < 0.05.

## 3. Results

### 3.1. Clinical and Anamnestic Characteristics

Clinical and anamnestic characteristics of the study groups presented in Table 2.

Comparative analysis demonstrated the following. Parity was significantly higher in patients with preeclampsia compared with those in the fetal loss syndrome group (*p* = 0.008). Number of pregnancies, frequency of spontaneous miscarriages, and VTE history did not differ significantly between groups (*p* > 0.05). Anemia was more frequently recorded in the preeclampsia group (*p* < 0.001). No statistically significant differences were found regarding other comorbidities involving the liver, hematologic system, kidneys, cardiovascular system, or autoimmune conditions.

**Figure 1:**
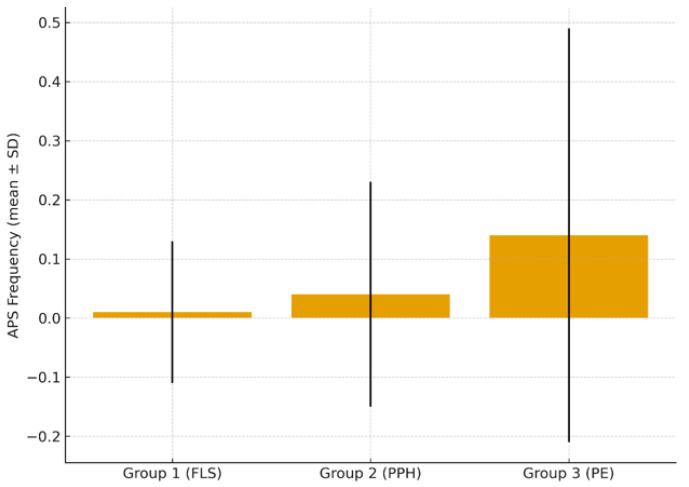
Frequency of APS across clinical groups.

Frequency of APS and non-criteria antiphospholipid antibodies. The mean APS detection rates were as follows: group 1—0.01 ± 0.12, group 2—0.04 ± 0.19, group 3—0.14 ± 0.35. A statistically significant increase in APS frequency was observed in the preeclampsia group (*p* = 0.032). Frequency of APS across clinical groups is presented on Figure 1.

### 3.2. Frequency of Thrombophilia-Related Genetic Polymorphisms in Women

The distribution of polymorphic genes associated with thrombophilia in women with fetal loss syndrome is presented in Table 3.

In the group of women with fetal loss syndrome, genetic polymorphisms mutations in the genes encoding coagulation factors—prothrombin *F2* and the Leiden genetic polymorphisms *F5*—were only found in the heterozygous state in 1.5% of cases; polymorphisms of the F7 gene were more common (18.9%). Polymorphisms of genes responsible for disorders in the fibrinolytic system were found in homo/heterozygous variants—the A allele of the *F13* gene in 21.2% and the A allele of the *FGB* gene in 26.5%. The C allele of the *ITGB3* gene (19.1%). More than half (62.1%) of women with fetal loss syndrome had the polymorphism T allele of the *ITGA2* gene (51.5% in hetero- and 10.6% in homozygous states) and the 5G allele of *PAI-1* (72.1%). In a group of women with a history of fetal loss syndrome and postpartum hemorrhage, prothrombin *F2* and *F5* Leiden genetic polymorphisms were found in a heterozygous state in 3.6% of cases, while *F7* gene polymorphisms were found in 17.9%. Polymorphisms of genes responsible for fibrinolytic disorders were also more common: *F13* (14.1%), *FGB* (25.0%), and *ITGB3* (10.7%). More than half of the women with fetal loss syndrome and postpartum hemorrhage had genetic polymorphisms alleles: the T allele of the *ITGA2* gene (64.3%) and the 5G allele of *PAI-1* (64.3%).

In a group of women with a history of fetal loss syndrome and preeclampsia, prothrombin *F2* and *F5* genetic polymorphisms were not detected; however, *F7* gene polymorphisms were found in a heterozygous state in 25.0% of cases. Polymorphisms of genes responsible for fibrinolytic disorders were also more common: *F13* (25.0%), *FGB* (33.3%), and *ITGB3* (16.7%). More than half of women with fetal loss syndrome and PE had polymorphisms alleles: the T allele of the *ITGA2* gene (72.3%) and the 5G allele of *PAI-1* (69.4%).

Statistically significant differences were found when comparing between the groups. Thus, heterozygous *F13* genetic polymorphisms were statistically more common in women with a history of preeclampsia compared to those with postpartum hemorrhage (the G/A genotype was detected in 22.2% versus 10.7%, *p* = 0.045), and heterozygous *ITGA2* genetic polymorphisms were also more common (the C/T genotype was detected in 66.7% versus 42.9%, *p* = 0.023). In the presence of a history of PPH, homozygous mutations of the *ITGA2* gene were statistically more frequently recorded (the T/T genotype was detected 2.6 times more often—21.4% versus 8.8% compared to the group with fetal loss syndrome, *p* = 0.022; 3.8 times more often—21.4% versus 5.6% compared to the group with PE, *p* = 0.022).

### 3.3. Folate-Cycle Gene Polymorphisms

The frequency of detection of polymorphic genes associated with folate cycle disorders in women with fetal loss syndrome is shown in Table 4.

In the general cohort of women with reproductive disorders, the *MTHFR:1286A > C* gene polymorphism was found in hetero- (34.8%) and homozygous states (9.1%); *MTR: 2756A > G* polymorphisms occurred in 36.4%, and *MTRR: 66 A > G* polymorphisms occurred in 69% (in heterozygous (47.0%) and homozygous (22.0%) states).

In the group of women with fetal loss syndrome, *MTHFR: 1286A > C* polymorphisms were statistically more common compared to other genes, and polymorphisms of genes responsible for folate cycle disorders were more common. *MTHFR: 1286A > C* polymorphisms occurred in heterozygous (41.2%) and homozygous (8.8%) states; *MTRR: 66 A > G* polymorphisms occurred in heterozygous (44.1%) and homozygous (20.6%) states.

Allele *C* of *MTHFR: 1286* gene was significantly more common in women with fetal loss syndrome (42.2% vs. 14.3% with PPH, *p* < 0.0003) and preeclampsia compared to the group with postpartum hemorrhage (38.9% versus 14.3%, (*p* < 0.001)).

The heterozygous form of the *MTR:2756* polymorphism was recorded significantly more often in women with preeclampsia (47.2%) compared to the group with fetal loss syndrome (27.9%, *p* < 0.026) and compared to PPH (25%, *p* < 0.009).

**Figure 2:**
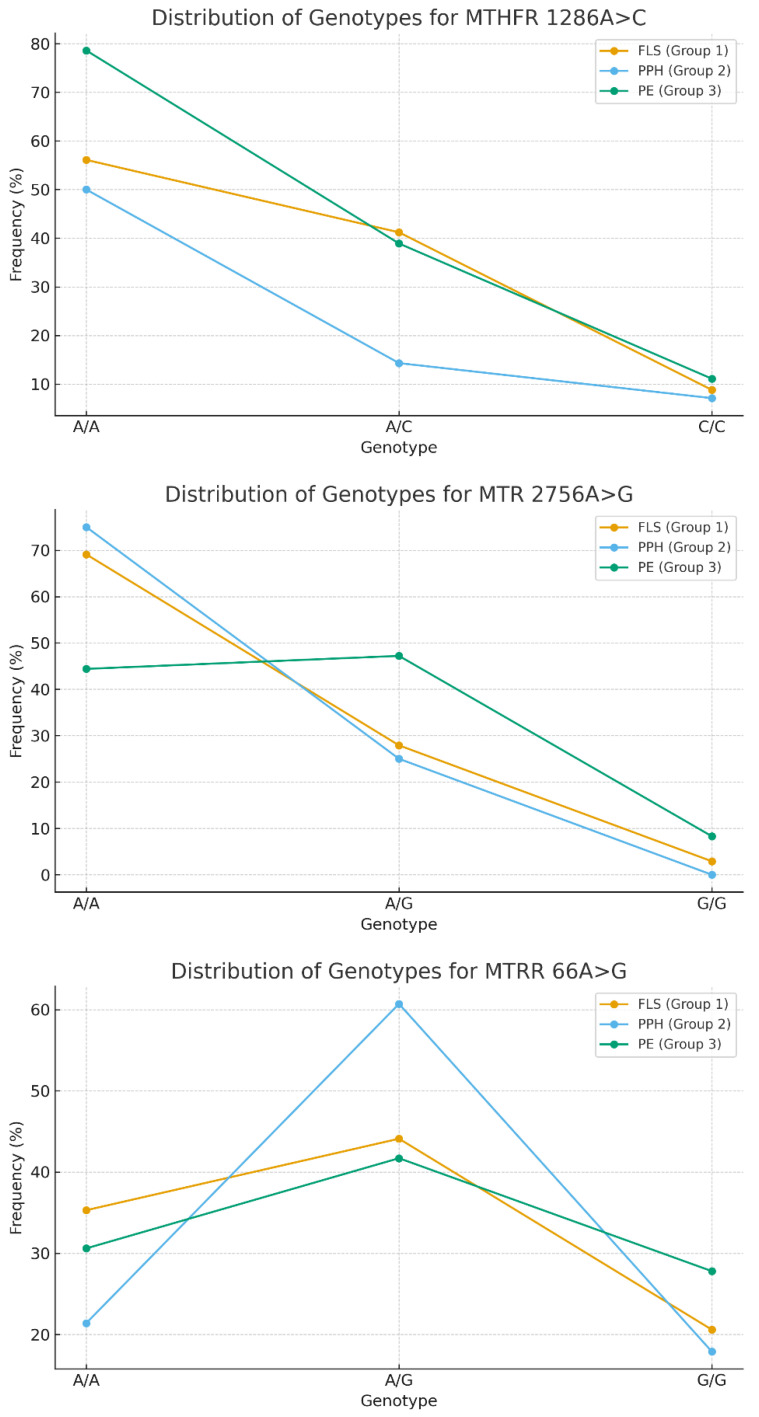
Distribution of genotypes.

Distribution of genotypes associated with folate metabolism disorders is presented on Figure 2.

Genetic polymorphisms in folate cycle genes lead to a decrease in the activity of the methionine synthase enzyme, and, as a consequence, to a disruption of homocysteine metabolism due to an increase in its concentration in the blood plasma. Hyperhomocysteinemia can cause early placental abruption, moderate and severe preeclampsia, fetal hypoxia, congenital pathology of the cardiovascular system and, as a result, termination of pregnancy. The frequency of the *A/G* and *G/G* genotypes of the *MTRR:66* polymorphism was comparable across all groups of women (*p* > 0.05).

For statistically significant differences between groups, the odds ratio (OR) and its 95% confidence interval (CI) were calculated and presented in Table 5.

The *MTHFR 1286A > C* (*A/C*) polymorphism demonstrated a protective effect against postpartum hemorrhage (PPH), suggesting a potential role of folate metabolism in the pathogenesis of postpartum bleeding (OR = 0.22, 95% CI 0.07–0.72, *p* = 0.008).

The *MTR 2756A > G* (*A/G*) genotype was associated with an increased risk of preeclampsia (PE) (OR = 2.63, 95% CI 1.11–6.25, *p* = 0.045), supporting the involvement of altered folate metabolism in the development of hypertensive pregnancy disorders.

Polymorphisms in *F13* and *ITGA2* showed no statistically significant associations with either PPH or PE, despite the presence of isolated non-significant trends, likely attributable to the limited sample size.

## 4. Discussion

A study of the clinical and anamnestic data of women with various pregnancy complications showed that in the group of patients with preeclampsia, the number of births was higher compared to patients with fetal loss syndrome (*p* = 0.008), and anemia (*p* < 0.001), the frequency of APS and non-criteria antiphospholipid antibodies (*p* = 0.032) were also recorded more often. The increased frequency of APS observed in patients with preeclampsia supports the current concept of APS as an independent risk factor for early-onset and severe preeclampsia, HELLP syndrome, and placental insufficiency [26,27,28,29]. The presence of antiphospholipid antibodies appears to potentiate the adverse effects of genetic thrombophilia, creating a pronounced prothrombotic background [27,28,29]. This underscores the necessity of targeted APS screening in women with hypertensive disorders of pregnancy, particularly in those with a history suggestive of thrombophilia [26,27,28,29].

The present study demonstrated that the genetic profile of thrombophilia in women with reproductive disorders varies depending on the clinical phenotype of pregnancy complications. First, the relatively low frequency of classical *F2* and *F5* gene polymorphisms aligns with evidence indicating that these variants are less prevalent in Asian populations compared with Caucasian populations [2,3,10,11]. At the same time, more frequent polymorphisms such as *F7*, *F13*, and *FGB* may play a substantial role in establishing a hypercoagulable state, particularly against the background of the physiological activation of coagulation during pregnancy [4,5,6].

Second, the observed associations with polymorphisms in platelet receptors *ITGA2* and *ITGB3* highlight the importance of the platelet component of hemostasis [7,8,9,12]. The increased prevalence of the heterozygous *ITGA2 C/T* genotype and the *T/C–T/T ITGA2* genotypes in patients with preeclampsia and postpartum hemorrhage may reflect enhanced platelet adhesion to the vascular endothelium, contributing to placental microthrombosis and impaired uteroplacental blood flow [7,8,12]. These findings are consistent with previous reports indicating a role of *ITGA2/ITGB3* in thromboembolic complications and adverse obstetric outcomes [9,12].

Third, the significant association between preeclampsia and the heterozygous *F13 G/A* genotype may be explained by alterations in fibrin clot structure and stability, which promote the development of microangiopathy and placental thrombosis [5,6,11]. Given the simultaneous increase in APS frequency in the preeclampsia group, one may hypothesize a synergistic interaction between genetic thrombophilia and autoimmune mechanisms in the pathogenesis of hypertensive disorders of pregnancy [11,26,27,28,29].

Particular attention should be given to folate-cycle polymorphisms. The higher frequency of the *MTHFR 1286C* allele in women with fetal loss syndrome and preeclampsia, as well as the association of the *MTR 2756 A/G* genotype with preeclampsia, suggests a potential role of hyperhomocysteinemia and impaired methylation in endothelial and placental damage [13,14,15,16]. These observations are consistent with studies demonstrating associations between *MTHFR/MTR* abnormalities and recurrent pregnancy loss, preeclampsia, and fetal growth restriction [13,14,15,16]. The distribution of *MTRR 66A > G* genotypes did not differ significantly between groups, which is consistent with findings from a large cohort study published in 2023 [15].

In the overall cohort, the *MTHFR c.1286A > C* gene polymorphism was detected in 34.8% of women in the heterozygous state and in 9.1% in the homozygous state. The *MTR c.2756A > G* polymorphism was identified in 36.4%, and *MTRR c.66A > G* in 69% of cases (47.0% heterozygotes and 22.0% homozygotes). These frequencies are consistent with data reported in Asian and Eurasian population studies [13,14,15].

The *C* allele of *MTHFR 1286* occurred significantly more often in women with fetal loss syndrome (42.2%) compared with those with postpartum hemorrhage (14.3%; *p* < 0.0003), and in patients with preeclampsia (38.9%) compared with the postpartum hemorrhage group (14.3%; *p* < 0.001). The *MTHFR 1286A > C* (*A/C*) genotype may be associated with a reduced risk of postpartum hemorrhage (OR = 0.22, 95% CI 0.07–0.72, *p* = 0.008). This polymorphism is known to be associated with mild hyperhomocysteinemia and impaired placentation [14,16]. The heterozygous *A/G* genotype of *MTR 2756* was significantly more common in women with preeclampsia (47.2%) compared with those with fetal loss syndrome (27.9%; *p* = 0.026) and with postpartum hemorrhage (25.0%; *p* = 0.009), which aligns with published evidence on the role of *MTR* in endothelial dysfunction [15,16]. The *MTHFR 2756A > G* (*A/G*) genotype may increase susceptibility to preeclampsia (OR = 2.63, 95% CI 1.11–6.25, *p* = 0.045).

Interestingly, the postpartum hemorrhage (PPH) group demonstrated a higher frequency of the *ITGA2 T/T* homozygous genotype, which may contribute to impaired platelet hemostasis in the postpartum period and predispose to massive hemorrhage in the presence of additional risk factors such as uterine atony or birth canal trauma [7,8,9,20,21]. Nevertheless, the association between PPH and genetic thrombophilia alone remains debatable and requires further investigation, taking into account obstetric factors [20,21,24,25].

### Limitations

The study has certain limitations, including the relatively small subgroup sample sizes, the absence of quantitative assessment of homocysteine levels, and the full spectrum of antiphospholipid antibodies in all participants. Future case–control studies are needed, including healthy individuals as a control group. However, the clear stratification by clinical phenotype (fetal loss syndrome, PPH, preeclampsia) and comprehensive genetic testing provide an important step toward personalized evaluation of thrombotic risk [24,25].

## 5. Conclusions

This study demonstrates phenotypic associations:

- Preeclampsia (PE): higher frequency of the *F13 G/A*, *ITGA2 C/T*, and *MTR 2756 A/G* genotypes.
- Postpartum hemorrhage (PPH): predominant association with the *ITGA2 T/T* genotype.

The higher prevalence of APS in PE suggests synergy between autoimmune and genetic thrombophilia mechanisms.

A wide range of genetic variants associated with thrombophilia were identified in women with reproductive disorders. Classic genetic polymorphisms, such as *F2 G20210A* and *F5* Leiden, were rare, while polymorphisms in *F7*, *F13*, *FGB*, *ITGA2*, *ITGB3*, and *PAI-1* were significantly more common. This suggests that the genetic architecture of thrombophilia in the study population is largely shaped by non-classical markers affecting the coagulation, platelet, and fibrinolytic pathways.

Patients with preeclampsia demonstrated a significantly higher frequency of the *F13 G/A*, *ITGA2 C/T*, and *MTR 2756 A/G* genotypes compared with women with postpartum hemorrhage. This profile reflects a combination of impaired fibrin formation, increased platelet reactivity, and folate-dependent endothelial dysfunction, suggesting a multifactorial contribution of genetic thrombophilia to the pathogenesis of hypertensive disorders of pregnancy.

Postpartum hemorrhage was associated with an increased prevalence of the *ITGA2 T/T* genotype, which may indicate specific platelet-hemostasis characteristics and serve as a potential genetic marker of severe PPH risk. These findings highlight the need for further investigation into platelet-receptor polymorphisms in the pathogenesis of atonic and mixed-type hemorrhages.

The frequency of obstetric antiphospholipid syndrome was statistically higher in patients with preeclampsia than in those with fetal loss syndrome. This supports the key role of APS in early and severe preeclampsia, placental insufficiency, and related perinatal complications, and underscores the importance of active screening for antiphospholipid antibodies in high-risk patients. The identified genotypes may serve as promising potential marker of preeclampsia and postpartum hemorrhage.

## Figures and Tables

**Table 1:** Detection site information.

Gene Name	RS Number	Base Change	Fluorescent Label
*F2* (*Prothrombin*)	*rs1799963*	c.*97G > A	FAM
*F5* (*Factor V Leiden*)	*rs6025*	*c.1601G > A*	FAM
*F7 (Factor VII)*	*rs6046*	*c.1172G > A*	FAM
*F13 (Factor XIII)*	*rs5985*	*c.103G > A*	HEX
*FGB* (*Fibrinogen*)	*rs1800790*	*g.4577G > A*	HEX
*ITGA2* (*Platelet Collagen Receptor*)	*rs1126643*	*c.759C > T*	HEX
*ITGB3* (*Glycoprotein IIIa*)	*rs5918*	*c.176G > A*	FAM
*PAI-1* (*Plasminogen Activator Inhibitor-1*)	*rs1799762*	*g.4332dup*	HEX
*MTHFR* (*Methylenetetrahydrofolate Reductase Enzyme*)	*rs1801131*	*c.1286A > C*	HEX
*MTR* (*Methionine Synthase*)	*rs1805087*	*c.2756A > G*	FAM
*MTRR* (*Methionine Synthase Reductase*)	*rs1801394*	*c.66A > G*	FAM

**Table 2:** Clinical and anamnestic characteristics of the study groups.

Parameter	Group 1		Group 2		Group 3		*p*		
	Mean	SD	Mean	SD	Mean	SD	1 and 2	1 and 3	2 and 3
Number of births	1.8	1.11	2.1	1.46	2.4	1.32	0.319	0.009 *	0.294
Number of pregnancies	4.6	1.34	4.6	1.79	4.6	1.32	0.936	1	0.94
Miscarriages	2.2	0.98	1.9	1.36	2.2	0.74	0.279	0.678	0.402
History of VTE	0.1	0.24	0.0	0.19	0.0	0.17	0.664	0.449	0.824
Infection (STI)	0.4	0.50	0.4	0.50	0.4	0.49	0.929	0.424	0.573
Surgery	0.3	0.45	0.3	0.48	0.0	0.17	0.705	0.001 *	0.004 *
Obesity	0.2	0.36	0.2	0.39	0.2	0.40	0.727	0.61	0.919
Uterine pathology	0.2	0.36	0.1	0.00	0.1	0.28	0.358	0.267	0.513
Diabetes mellitus	0	0.17	0.1	0.19	0.1	0.35	0.337	0.075	0.301
Anemia	0.3	0.44	0.0	0.51	0.5	0.51	0.061	0.005 *	0.0002 *
Liver disease	0	0	0.5	0	0	0.17	0	0.274	1.428
Blood diseases	0	0.21	0	0	0	0.17	0.115	0.787	0.274
Kidney disease	0	0	0	0	0.1	0.35	0	0.0186	0.0186
Heart disease	0	0	0	0	0	0.17	0	0.274	0.274
Thrombocytopenia	0.1	0.24	0	0	0.1	0.23	0.04	1	0.119
Thrombophilia/APS	0.	0.12	0	0.19	0.1	0.35	0.442	0.032	0.141
Autoimmune diseases	0	0.12	0.1	0.26	0.2	0.38	0.254	0.015	0.209

Note. * *p* < 0.05.

**Table 3:** Frequency of thrombophilia-related genetic polymorphisms in women with fetal loss syndrome.

Gene Names	Genotypes	Frequency of Genotype Detection, %			*p*		
		FLS (1)	PPH (2)	AG (3)	1 and 2	1 and 3	2 and 3
*F2*	*G/G*	67 (98.5)	27 (96.4)	36 (100.0)	0.880	0.917	0.799
	*G/A*	1 (1.5)	1 (3.6)	0 (0)	0.349	0.225	0.059
	*A/A*	0 (0)	0 (0)	0 (0)	-	-	-
*F5*	*G/G*	67 (98.5)	27 (96.4)	36 (100.0)	0.880	0.917	0.799
	*G/A*	1 (1.5)	1 (3.6)	0 (0)	0.349	0.225	0.059
	*A/A*	0 (0)	0 (0)	0 (0)	-	-	-
*F7*	*G/G*	57 (83.8)	23 (82.1)	27 (75.0)	0.896	0.484	0.569
	*G/A*	11 (16.2)	5 (17.9)	9 (25.0)	0.773	0.169	0.275
	*A/A*	0 (0)	0 (0)	0 (0)	-	-	-
*F13*	*G/G*	53 (77.9)	24 (85.7)	27 (75.0)	0.543	0.812	0.398
	*G/A*	14 (20.6)	3 (10.7)	8 (22.2)	0.078	0.803	0.045 *
	*A/A*	1 (1.5)	1 (3.6)	1 (2.8)	0.349	0.526	0.753
*FGB*	*G/G*	52 (76.5)	21 (75.0)	24 (66.7)	0.905	0.413	0.484
	*G/A*	15 (22.1)	6 (21.4)	12 (33.3)	0.924	0.130	0.108
	*A/A*	1 (1.5)	1 (3.6)	0 (0)	0.349	0.225	0.059
*ITGA2*	*C/C*	30 (44.1)	10 (35.7)	10 (27.8)	0.347	0.054	0.319
	*C/T*	32 (47.1)	12 (42.9)	24 (66.7)	0.658	0.066	0.023 *
	*T/T*	6 (8.8)	6 (21.4)	2 (5.6)	0.022 *	0.389	0.002 *
*ITGB3*	*T/T*	55 (80.9)	25 (89.3)	30 (83.3)	0.519	0.848	0.651
	*T/C*	13 (19.1)	3 (10.7)	6 (16.7)	0.124	0.682	0.255
	*C/C*	0 (0)	0 (0)	0 (0)	-	-	-
*PAI-1*	*4G/4G*	19 (27.9)	10 (35.7)	11 (30.6)	0.330	0.732	0.526
	*5G/4G*	35 (51.5)	14 (50.0)	16 (44.4)	0.884	0.473	0.568
	*5G/5G*	14 (20.6)	4 (14.3)	9 (25.0)	0.286	0.513	0.087

* *p* < 0.05.

**Table 4:** Frequency of occurrence of genetic polymorphisms associated with folate metabolism disorders in women with fetal loss syndrome.

Gene Names	Genotypes	Frequency of Genotype Detection, %					*p*		
		Total		FLS (1)	PPH (2)	PE (3)	1 and 2	1 and 3	2 and 3
*MTHFR:* 1286A > C	*A/A*	74	56.1	34 (50.0)	22 (78.6)	18 (50.0)	0.012 *	1.000	0.012 *
	*A/C*	46	34.8	28 (41.2)	4 (14.3)	14 (38.9)	0.0003 *	0.798	0.001 *
	*C/C*	12	9.1	6 (8.8)	2 (7.1)	4 (11.1)	0.674	0.608	0.353
*MTR:* 2756A > G	*A/A*	84	63.6	47 (69.1)	21 (75.0)	16 (44.4)	0.624	0.021 *	0.005 *
	*A/G*	43	32.6	19 (27.9)	7 (25.0)	17 (47.2)	0.686	0.026 *	0.009 *
	*G/G*	5	3.8	2 (2.9)	0 (0)	3 (8.3)	0.086	0.108	0.004 *
*MTRR:* 66A > G	*A/A*	41	31	24 (35.3)	6 (21.4)	11 (30.6)	0.066	0.559	0.206
	*A/G*	62	47.0	30 (44.1)	17 (60.7)	15 (41.7)	0.105	0.791	0.060
	*G/G*	29	22.0	14 (20.6)	5 (17.9)	10 (27.8)	0.660	0.301	0.142

* *p* < 0.05.

**Table 5:** Odds ratio (OR) and 95% confidence interval (CI) in women with PPH and PE.

Genotypes	Alleles	PPH		PE	
		OR (95% CI)	*p*	OR (95% CI)	*p*
F13	G/A	0.47 (0.12–1.8)	0.378	1.12 (0.42–3.0)	0.805
	A/A	2.21 (0.13–36.81)	0.536	1.96 (0.12–32.62)	1
ITGA2	C/T	1.12 (0.42–2.99)	1	2.25 (0.92–5.48)	0.086
	T/T	3.0 (0.79–11.44)	0.153	1.0 (0.17–5.77)	1
Folate Metabolism Disorders					
MTHFR: 1286A > C	A/C	0.22 (0.07–0.72)	0.008 *	0.94 (0.4–2.23)	1
	C/C	0.52 (0.1–2.79)	0.699	1.26 (0.31–5.05)	0.733
MTR: 2756A > G	A/G	0.82 (0.3–2.26)	0.804	2.63 (1.11–6.25)	0.045 *
	G/G	0.44 (0.02–9.6)	1	4.41 (0.67–28.79)	0.129

* *p* < 0.05.

## Data Availability

The original contributions presented in this study are included in the article. Further inquiries can be directed to the corresponding author.
